# Impact of neuronal heterogeneity on correlated colored noise-induced synchronization

**DOI:** 10.3389/fncom.2013.00113

**Published:** 2013-08-21

**Authors:** Pengcheng Zhou, Shawn D. Burton, Nathaniel N. Urban, G. Bard Ermentrout

**Affiliations:** ^1^Program in Neural Computation, Carnegie Mellon UniversityPittsburgh, PA, USA; ^2^Center for the Neural Basis of CognitionPittsburgh, PA, USA; ^3^Department of Biology, Carnegie Mellon UniversityPittsburgh, PA, USA; ^4^Department of Mathematics, University of PittsburghPittsburgh, PA, USA

**Keywords:** synchrony, correlation, colored noise, heterogeneity, neural oscillators, phase-response curve

## Abstract

Synchronization plays an important role in neural signal processing and transmission. Many hypotheses have been proposed to explain the origin of neural synchronization. In recent years, correlated noise-induced synchronization has received support from many theoretical and experimental studies. However, many of these prior studies have assumed that neurons have identical biophysical properties and that their inputs are well modeled by white noise. In this context, we use colored noise to induce synchronization between oscillators with heterogeneity in both phase-response curves and frequencies. In the low noise limit, we derive novel analytical theory showing that the time constant of colored noise influences correlated noise-induced synchronization and that oscillator heterogeneity can limit synchronization. Surprisingly, however, heterogeneous oscillators may synchronize better than homogeneous oscillators given low input correlations. We also find resonance of oscillator synchronization to colored noise inputs when firing frequencies diverge. Collectively, these results prove robust for both relatively high noise regimes and when applied to biophysically realistic spiking neuron models, and further match experimental recordings from acute brain slices.

## 1. Introduction

Synchronization of neural oscillators is thought to play a critical role in sensory, motor, and cognitive processes (Sanes and Donoghue, 1993; Fries et al., 2001; Wang, 2010). In many networks, synchronization is achieved via direct coupling such as through gap junctions and chemical synapses. However, there are several systems (notably, the mammalian olfactory bulb) where the mode of coupling is less clear and neural synchrony is hypothesized to arise from partially correlated presynaptic inputs (Galán et al., 2006; Marella and Ermentrout, 2010). Indeed, in non-oscillatory networks of neurons, such correlated input is largely responsible for the output correlations of the neurons (de la Rocha et al., 2007). Thus, a natural question is: how do the properties of neurons and networks alter output correlations for a given degree of input correlation? At small input correlations, output and input correlations can be regarded as linearly proportional; this ratio is called the *susceptibility* (Shea-Brown et al., 2008). For example, (de la Rocha et al., 2007) showed that the susceptibility depends on the background firing rate of the neuron. For some model systems, this susceptibility can be computed using linear response theory (which assumes small perturbations around the stationary state).

When neurons fire regularly, they can be regarded as noisy nonlinear oscillators and, as such, there are many mathematical techniques available for their analysis. In particular, the *phase-response curve* (PRC) provides a compact and useful characterization of the responses of a nonlinear oscillator to external perturbations. The PRC describes the shift in timing of, say, an action potential as a function of the timing of the input relative to the last action potential. In several studies, we have described the theoretical relationship between the shape of the PRC and the ability of *identical* neurons to transfer partially synchronized activity (Marella and Ermentrout, 2008; Abouzeid and Ermentrout, 2009). In these studies, the only source of heterogeneity considered between neural oscillators was their unshared (uncorrelated) inputs, which consisted of white noise. Recently, we extended these methods to cases in the low noise limit in which the oscillators were not identical and showed how heterogeneity in intrinsic properties could significantly degrade the output correlation in pairs receiving common inputs (Burton et al., 2012).

In this study, we extend this theory to include colored noise inputs and, further, report some surprising effects of heterogeneity. First, we derive a set of equations for the distribution of phase differences for pairs of heterogeneous oscillators driven by a partially correlated Ornstein-Uhlenbeck (OU) process (low-pass filtered noise). We next show that the theory developed for phase reduced models works well with a conductance-based biophysical model. We then show that, quite surprisingly, at low input correlations, heterogeneity can sometimes produce higher output correlations than the homogeneous case. That is, consider two distinct oscillators, A and B, such that the AA pair has a small susceptibility and the BB pair a larger susceptibility. Then, at low correlations, the susceptibility of the AB pair can sometimes exceed that of the AA pair. We confirm this somewhat counterintuitive prediction with recordings from regularly firing mitral cells of the main olfactory bulb. In addition to heterogeneity in response properties, neurons can fire at different frequencies, and such frequency differences can significantly impact correlated-noise induced synchronization (Markowitz et al., 2008; Burton et al., 2012). Here, we find that for some frequency differences between oscillators, there is an optimal time scale of correlated noise that will maximally synchronize the oscillators. We do not see this effect when the oscillators have the same frequency.

## 2. Materials and methods

### 2.1. Phase reduction model

In Appendix, we provide a brief overview of how to reduce a general weakly perturbed limit cycle to a single differential equation for the phase of the cycle. If we assume that the original limit cycle represents repetitive firing of a single compartment neuron model that is driven by a noisy current, *I*(*t*), then we obtain:

(1) $$\frac{d\theta }{dt}=1+\epsilon \Delta (\theta )I(t)/C_{m}$$

where *C*_*m*_ is the membrane capacitance, θ is the phase (or, typically, the time since the last spike), and Δ(θ) is the PRC of the neuron. The PRC describes the phase-dependent shift in the spike times of an oscillator receiving small perturbations. It is readily measured in neurons and other biological oscillators (Torben-Nielsen et al., 2010) and provides a compact representation of the effects of stimuli on the timing of action potentials. Δ(θ) has dimensions of milliseconds per millivolt; that is, the shift in timing of the next action potential per millivolt perturbation of the potential. Mathematically, for a given model, Δ(θ) is found by solving a certain differential equation (see Appendix). It is a periodic function of phase and, with no loss in generality, we can normalize the period to be 2π for simplicity.

### 2.2. Stationary density

Given the reduced model (Equation 1), we can now turn to the main question at hand, which is: how do oscillating heterogeneous neurons transfer correlations? We will consider two types of heterogeneity: differences in the PRC shapes and differences in natural frequencies. We drive the oscillators with correlated filtered noise. After reduction to phase variables, we obtain:

(2) $$\theta_{1}^{\prime }=1+\epsilon \Delta_{1}(\theta_{1})x$$

(3) $$\theta_{2}^{\prime }=1+\epsilon \Delta_{2}(\theta_{2})y+\epsilon^{2}\omega$$

(4) $$x^{\prime }=-x/\tau +\xi_{x}/\sqrt{\tau }$$

(5) $$y^{\prime }=-y/\tau +\xi_{y}/\sqrt{\tau }$$

θ_1_ and θ_2_ are the phases of two oscillators, and Δ_1_(θ) and Δ_2_(θ) are PRCs of the two oscillators. Without loss of generality, we set the natural frequency of one oscillator to 1. The parameter ω then determines the magnitude of the difference in natural frequencies between the two oscillators. ϵ ≪ 1, thus the noise is weak and the frequency difference is small. The processes *x* and *y* are generated by an OU process with the same time constant τ. ξ_*x*_ and ξ_*y*_ are two correlated white noise processes, i.e., 〈ξ_*x*_(*t*)ξ_*x*_(*t*′)〉 = δ(*t* − *t*′), 〈ξ_*y*_(*t*)ξ_*y*_(*t*′)〉 = δ(*t* − *t*′), 〈ξ_*x*_(*t*)ξ_*y*_(*t*′)〉 = *c*δ(*t* − *t*′), where *c* is the degree of correlation.

We remark that the allowable frequency difference is *O*(ϵ^2^), which seems considerably smaller than the magnitude of the noise, which is ϵ. However, as the noise has zero mean, what matters is the variance of the noise, which has magnitude ϵ^2^. Thus, the scales of both the frequency difference and the synchronizing inputs (correlations in the noise) are similar. If the frequency differences are larger, then no synchronization is possible.

Our goal is to compute the stationary distribution of the phase difference between two neurons since this will enable us to compute various measures of correlation and synchrony. Thus, some variable substitution will be helpful: θ = θ_1_, ϕ = θ_2_ − θ_1_. Therefore, ϕ is the phase difference between the two oscillators. With this change of variables, the equations are:

(6) $$\theta^{\prime }=1+\epsilon \Delta_{1}(\theta )x$$

(7) $$\phi^{\prime }=\epsilon [\Delta_{2}(\theta +\phi )y-\Delta_{1}(\theta )x]+\epsilon^{2}\omega$$

and *x*, *y* are as above. Let ρ(*x*, *y*, θ, ϕ, *t*) represent the probability density function at time *t*:

(8) $$\begin{array}{l} Pr(X(t)\in (x,x+dx),Y(t)\in (y,y+dy),\Theta (t)\in (\theta ,\theta +d\theta ),\\ \;\Phi \text{​}(t)\in (\phi ,\phi +d\phi ))=\rho (x,y,\theta ,\phi )dxdyd\theta d\phi \end{array}$$

We denote the stationary density (long-time behavior as *t* → ∞) as ρ_*ss*_(*x*, *y*, θ, ϕ).

Our goal is to compute the probability density of the phase difference between the two oscillators, *R*(ϕ), which is:

(9) $$R(\phi ):=\int_{-\infty }^{\infty }\int_{-\infty }^{\infty }\int_{0}^{2\pi }\rho_{ss}(x,y,\theta ,\phi )\;dxdyd\theta$$

If the oscillators are perfectly synchronized, then *R*(ϕ) will be a delta function centered at ϕ = 0. If the oscillators are completely independent, then *R*(ϕ) = 1/(2π). In Appendix, we show that *R*(ϕ) satisfies a simple first order boundary value problem (BVP). We present the exact equation for this in Results.

### 2.3. Order parameter

Once we get the distribution of phase differences, *R*(ϕ), we need a number to estimate the synchronization, which means the sharpness of this distribution. In this study, we use an order parameter (OP) to do this. We define:

(10) $$\begin{array}{l} \text{OP}=\sqrt{C^{2}+S^{2}}\\ \;C=\int_{0}^{2\pi }R(\phi )\cos(\phi )d\phi \\ \;S=\int_{0}^{2\pi }R(\phi )\sin(\phi )d\phi \\ \;\theta =\text{atan2}(C,S) \end{array}$$

OP is a representation of sharpness and θ is the estimation of the peak position. For certain types of heterogeneity, *R*(ϕ) is peaked at ϕ = 0; in this case, we can show that the cross correlation of the spike times is (*R*(0) − 1/(2π))/(2π) (Burton et al., 2012). However, OP provides a better global measure of the synchrony and is not dependent on the peak being centered at 0; we will therefore use OP in our current results.

### 2.4. Morris-lecar model

The Morris-Lecar (ML) model (Rinzel and Ermentrout, 1989) is a simplified two-dimensional system membrane model that we use to compare the phase models with a full biophysical model:

(11) $$\begin{array}{l} C\frac{dV_{1}}{dt}=I_{1}-g_{L}(V_{1}-V_{L})-g_{K}w_{1}(V_{1}-V_{K})\\ \;-g_{Ca}m_{\infty }(V_{1})(V_{1}-V_{Ca})+\sigma x \end{array}$$

(12) $$\frac{dw_{1}}{dt}=\phi \frac{w_{\infty }(V_{1})-w_{1}}{\tau_{w}(V_{1})}$$

(13) $$\begin{array}{l} C\frac{dV_{2}}{dt}=I_{2}-g_{L}(V_{2}-V_{L})-g_{K}w_{2}(V_{2}-V_{K})\\ \;-g_{Ca}m_{\infty }(V_{2})(V_{2}-V_{Ca})+\sigma x \end{array}$$

(14) $$\frac{dw_{2}}{dt}=\phi \frac{w_{\infty }(V_{2})-w_{2}}{\tau_{w}(V_{2})}$$

(15) $$x^{\prime }=-x/\tau +\xi_{x}/\sqrt{\tau }$$

(16) $$y^{\prime }=-y/\tau +\xi_{y}/\sqrt{\tau }$$

with 〈 ξ_1_(*t*), ξ_1_(*t*′)〉 = δ(*t* − *t*′), 〈 ξ_2_(*t*), ξ_2_(*t*′)〉 = δ(*t* − *t*′), and 〈ξ_1_(*t*), ξ_2_(*t*′)〉 = *c*δ(*t* − *t*′), *c* ∈ [0, 1]. The auxiliary functions are:

(17) $$m_{\infty }(V)=0.5\cdot (1+\tanh((V-V_{a})/V_{b}))$$

(18) $$w_{\infty }(V)=0.5\cdot (1+\tanh((V-V_{c})/V_{d}))$$

(19) $$\tau_{w}(V)=\frac{1}{\cosh((V-V_{c})/(2V_{d}))}$$

The parameters used in this paper are: *V*_*K*_ = −84 mV, *V*_*L*_ = −60 mV, *V*_*Ca*_ = 120 mV, $g_{K}=8\frac{mS}{cm^{2}}$, $g_{L}=2\frac{mS}{cm^{2}}$, $g_{Ca}=4\frac{mS}{cm^{2}}$, $C=20\frac{\mu F}{cm^{2}}$, *V*_*a*_ = −1.2 mV, *V*_*b*_ = 18 mV, *V*_*c*_ = 2 mV, and *V*_*d*_ = 30 mV. *I*_1_, *I*_2_ and ϕ_1_, ϕ_2_ vary for each figure.

To get the phase from the noisy voltage signal generated by the ML model, we first apply the Hilbert transform (HT) to *V*(*t*) which allows us to get a phase. However, the phase is not uniform as it is not a temporal phase. We then map the HT phase to a temporal phase on the noise-free limit cycle which gives a uniform phase-distribution as required by the theory. This allows us to estimate *R*(ϕ) for the biophysical model, where ϕ here is the phase difference between two ML model neurons that are driven with partially correlated noise.

In some of the figures, we simulate the phase-reduced dynamics for the ML model. To do this, we must compute the infinitesimal PRC, Δ_*ML*_(θ). As described in Appendix, the PRC for the model is the voltage component of the solution to the adjoint equation (Equation 32). The software package XPP (Ermentrout, 2002) includes an algorithm for computing the adjoint solution for an exponentially stable limit cycle, so we simply compute various limit cycles (say with very different parameters but similar periods) and then compute Δ_*ML*_(θ) for those specific parameters. We save the result as a lookup table and then numerically solve the phase equation.

### 2.5. Numerics

To get solutions to the stochastic phase and membrane equations, we use the Euler-Murayama method. We solve the BVP for the stationary phase difference density using a custom BVP solver written in MATLAB. All codes are available by request.

## 3. Results

### 3.1. Approximation of the phase difference density

Oscillators driven with a correlated fluctuating signal will exhibit a degree of synchronization that depends on the size of the signal, the strength of correlation, and the similarity of the two oscillators. Thus, for example, identical oscillators driven by small enough identical white noise will synchronize perfectly (Pikovsky et al., 1997; Teramae and Tanaka, 2004). The rate at which these identical oscillators synchronize depends on the properties of the noise - in particular, its autocorrelation (Nakao et al., 2007; Goldobin et al., 2010). In general, and especially in biological systems, there will be a great deal of heterogeneity in any pair of oscillators. For example, for neurons, there is always some source of independent noise so that the input correlation is always less than 1. The neurons may also be firing at slightly different frequencies. Finally, even if the neurons are adjusted to fire at the same frequency, their distribution of ion channels can be very different and, thus, their response to correlated signals can be quite different (Burton et al., 2012). If the fluctuating inputs are sufficiently small, then any stable limit cycle oscillator can be reduced to a so-called phase model where the dynamics are characterized by a single variable, the phase, such that the firing is considered to occur at a phase of 0 and the time between spikes is mapped onto an angle between zero and 2π. Here, we consider driven pairs of heterogeneous oscillators that receive partially correlated filtered noise. As our main examples come from neuroscience, we assume that the external inputs are implemented as currents, in which case the phase model for the pair of neural oscillators has the form:

$$\begin{array}{l} \theta_{1}^{\prime }=1+\epsilon \Delta_{1}(\theta_{1})x(t)\\ \theta_{2}^{\prime }=1+\epsilon^{2}\omega +\epsilon \Delta_{2}(\theta_{2})y(t) \end{array}$$

where *x*(*t*) and *y*(*t*) are OU processes with the same time constant, τ, and with correlation *c*; Δ_1, 2_(θ) are the PRCs for the two oscillators; ϵ is a small positive parameter (characterizing the magnitude of the fluctuations); and ω accounts for the frequency difference in the unperturbed oscillators (see Materials and Methods, Equations 2–5). We are primarily interested in the distribution of the phase difference, ϕ: = θ_2_ − θ_1_. In the Appendix (Equation 62), we show that *R*(ϕ), the probability density function for the phase difference, satisfies a simple BVP:

$$\begin{array}{l} \frac{d}{d\phi }\{[c\cdot g(\phi )-C_{1}]R(\phi )\}+(4\pi \omega -C_{2})R(\phi )=K\\ \;R(\phi )=R(\phi +2\pi )\\ \;g(\phi )=g(\phi +2\pi )\\ \;\int_{-\pi }^{\pi }R(\phi )d\phi =1\\ \;K=2\omega -\frac{C_{2}}{2\pi } \end{array}$$

**Figure 1:**
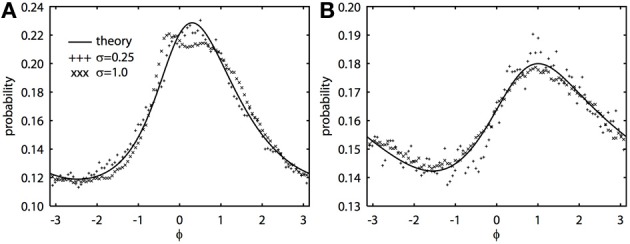
**Novel analytical theory of correlated colored noise-induced synchronization of heterogeneous oscillators matches Monte Carlo simulations for low to moderate levels of noise.** Stationary phase difference density is shown as computed from the solution of the BVP and through Monte Carlo simulation from *t* = 1000 to *t* = 201000 in steps of 0.05. Monte Carlo data binned into 100 bins between −π and π. There is a frequency difference of ϵ^2^/2 where ϵ is the magnitude of the noise. Here Δ_*j*_(θ_*j*_) = sin(*a*_*j*_) − sin(θ_*j*_ + *a*_*j*_) + *b*_*j*_ sin(2θ_*j*_), where *j* = 1, 2 for two oscillators. **(A)** τ = 1, *a*_1_ = 0.1, *a*_2_ = 0.6, *b*_1_ = 0.32, *b*_2_ = 0.3, and *c* = 0.8. **(B)** τ = 0.25, *a*_1_ = *a*_2_ = 0.5, *b*_1_ = *b*_2_ = 0.3, and *c* = 0.5.

The 2π−periodic function *g*(ϕ) and the constants, *C*_1, 2_, depend in a complicated way on the forms of the PRCs and the time constant of the noise, τ (see Appendix). However, all quantities can be found by integrating elementary functions. If the oscillators have the same PRC, then *C*_2_ = 0 and *g*(ϕ) is even symmetric. If the oscillators have the same frequency, then ω = 0. When both *C*_2_ and ω vanish, we can immediately solve the BVP, yielding *R*(ϕ) = *N*/(*C*_1_ − *cg*(ϕ)), where *N* is a normalization constant so that the integral is 1. This is the result found in Marella and Ermentrout (2008) for white noise, but is clearly also true for colored noise. When the oscillators are identical and there is no difference in frequencies, the phase difference density is symmetric and always peaks at 0. However, when ω − *C*_2_ is nonzero, the peak of the phase difference density will generally be offset. We note that ϵ does not appear in the expression for *R*(ϕ), which says that the phase difference density is, to a first approximation, independent of the amplitude of the noise. Figure 1 shows typical results comparing the perturbation calculation and the simulation of the Langevin equation. In Figure 1A, at fairly high noise ϵ = 1, there is some distortion at the peak of the distribution, but as predicted from the theory, the distribution magnitude is largely independent of the magnitude of the fluctuations. Figure 1B shows a similar simulation, but the correlation of noise is lower (*c* = 0.5 vs. *c* = 0.8), the noise is faster (τ = 0.25 vs. τ = 1), and the PRCs are identical. In this case, even the higher noise simulations match the theory. We once again emphasize that the perturbation expansion requires a small value of ϵ, but clearly, the simulations show that ϵ can be nearly 1 and still yield good agreement.

We note that the density of the phase differences can be related to more conventional measures of correlation. In Burton et al. (2012), we showed that the spike time cross-correlation (CC) between a pair of weakly noisy oscillators is:

(20) $$\text{CC}(\tau )=\frac{1}{2\pi }\left[R(-\tau )-\frac{1}{2\pi }\right]$$

For example, if the oscillators are asynchronous, then they have a uniform phase difference density and the cross-correlation will be 0. This calculation confirms ones intuition that *different* neurons that receive correlated noise will have spike time cross-correlations that peak off-center.

**Figure 2:**
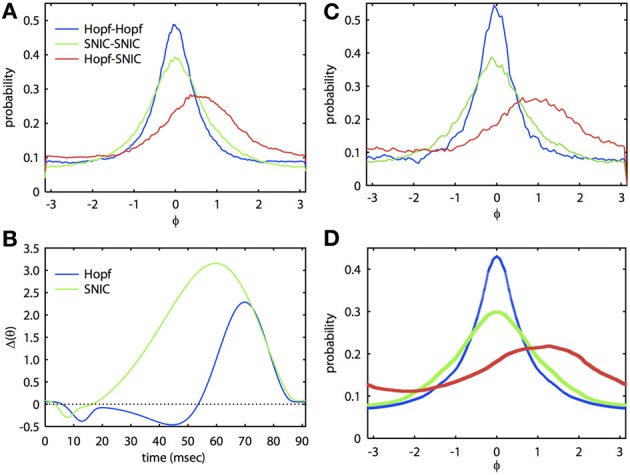
**Analytical theory accurately predicts synchronization of biophysically realistic spiking neuron models. (A)** Invariant phase difference density computed from the reconstructed phase of two ML model neurons receiving partially correlated colored noise (period is 91.25 ms, τ = 5 ms, *c* = 0.8). Three cases are illustrated with either identical (homogeneous) or mixed (heterogeneous) PRCs. The “Hopf” case corresponds to a set of parameters where the oscillatory activity arises via a HB and the “SNIC” case through a SNIC bifurcation (Rinzel and Ermentrout, 1989); see Appendix for parameters. **(B)** PRCs for the two cases. **(C)** Same as **(A)**, but using simulations of the phase reduced equations. **(D)** Solutions to the BVP using the PRCs from **(B)**.

Figure 2 shows that we can apply the theory through two levels of simplification. The ML system is a simple, biophysically realistic model for a spiking neuron (Rinzel and Ermentrout, 1989). With different choices of parameters, the onset to oscillatory behavior can be either through a Hopf bifurcation (HB) or a saddle-node on an invariant circle (SNIC) bifurcation. The PRCs that result from these distinct bifurcations are often quite different (Brown et al., 2004; Izhikevich, 2007) and thus have quite different synchronization properties. In Figure 2, we tune the ML model so that each cell has the same frequency but the parameters are quite different and so the PRCs are different (see Figure 2B). In Figure 2C, we show the results of a Monte Carlo simulation in which the biophysical model is driven by correlated noise. Phase is reconstructed from the voltage traces using a Hilbert transform and from these, we obtain phase difference histograms. In this figure, the correlation *c* is 0.8, τ = 5 ms, and the natural period of the oscillation is 91.25 ms. For the same degree of correlation, two HB oscillators are much better at synchronizing than are two SNIC oscillators. This result is consistent with the theory developed in Marella and Ermentrout (2008) for white noise and also for spike time correlations over fast timescales (i.e., spike synchrony) (Barreiro et al., 2010). At this high correlation, the heterogeneous HB-SNIC pair shows greatly reduced synchrony from either of the homogeneous cases and a shift in the peak *even though there is no frequency difference*. Figure 2B shows the two PRCs that were determined using the adjoint method. We then used these PRCs to compute the invariant densities for the corresponding phase reduced models. The invariant density is a function that describes the distribution of phase differences of the two neurons over some time interval consisting of many cycles. Thus, the peak of the invariant density indicates the most likely phase difference, and a large peak at zero phase difference would indicate that the two neurons are well synchronized. Comparison between Figures 2A,C shows excellent agreement. Finally, we substituted the numerically computed PRCs into our BVP and computed the invariant density. The result of this calculation is shown in Figure 2D. There are small differences in the amplitude, but the shapes and the shift of the densities in the heterogeneous case are almost identical. Thus, through two levels of reduction (first, from the full model to the phase model, and second, from the Langevin phase model to the approximate invariant density), we see that our analytical method works very well at estimating the invariant density of phase differences between neural oscillators.

### 3.2. PRC heterogeneity

Our approximation of the invariant density, while requiring that we solve a BVP, allows us to explore the effects of heterogeneity much faster than simulating the appropriate Monte Carlo system. Thus, we will use this method to explore the effects of PRC heterogeneity, frequency differences, and the color of the noise on the ability of oscillators to synchronize. One simple global measure of synchrony/correlation for systems whose natural dynamics are periodic is the circular variance, σ_*circle*_ = 1 − OP, where we define an order parameter (OP) (see Materials and Methods, Equation 10):

$$\text{OP}={\left[{\left(\int_{-\pi }^{\pi }R(\phi )\cos\phi \;d\phi \right)}^{2}+{\left(\int_{-\pi }^{\pi }R(\phi )\sin\phi \;d\phi \right)}^{2}\right]}^{\frac{1}{2}}$$

For a flat distribution, OP = 0 (σ_circle_ = 1) and for a delta function distribution, OP = 1 (σ_circle_ = 0). The OP is a commonly used measure for the degree of synchronization between two oscillators (Kuramoto, 2003).

**Figure 3:**
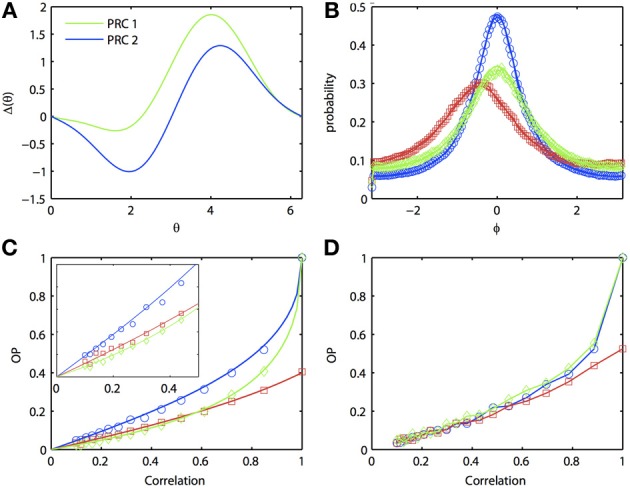
**Oscillator heterogeneity can enhance correlated noise-induced synchronization at low input correlations. (A)** Two PRCs with the form Δ_*j*_(θ_*j*_) = sin(*a*_*j*_) − sin(*a*_*j*_ + θ_*j*_) + *b*_*j*_ sin(2θ_*j*_), *a*_1_ = 0.1, *b*_1_ = 0.32, *a*_2_ = 0.6, and *b*_6_ = 0.3. **(B)** *R*(ϕ) with different combinations of PRCs. Blue: PRC 2-PRC 2; red: PRC 1-PRC 2; green: PRC 1-PRC 1. Solid lines are theoretical predictions from the solution to the BVP and open symbols are Monte Carlo simulation results (same notation applies in following figures). Parameters used: τ = 1 and *c* = 0.8. **(C)** Synchronization as input correlation varies from 0 to 1; inset shows magnification when *c* < 0.5. **(D)** Same as **(C)**, but using the ML model. Parameters used: *I*_1_ = 110, ϕ_1_ = 0.04616, *I*_2_ = 120, and ϕ_2_ = 0.04.

In general, one expects that the synchrony between two oscillators forced with correlated noise would be greatest if the oscillators are homogeneous. Certainly, if the inputs are identical (i.e., no independent or unshared noise), then identical oscillators will synchronize perfectly, while heterogeneous oscillators will not synchronize perfectly. That is, the phase density will not be a delta function. [See Burton et al. (2012) for a proof]. However, surprisingly, at low input correlations, it is possible for a heterogeneous pair of oscillators to produce greater synchrony than one (but not both) of the respective homogeneous pairs of oscillators. Figure 3 illustrates the behavior of two separate homogeneous pairs of oscillators (blue and green lines, respectively) as the input correlation varies from 0 to 1. A third, heterogeneous pair comprised of an oscillator from each homogeneous pair is shown in red. Figure 3A shows the two different PRCs; pairs of oscillators with the green PRC (“PRC 1-PRC 1”) produce weaker synchrony than pairs of oscillators with the blue PRC (“PRC 2-PRC 2”). This is demonstrated in Figure 3B, where the correlation is set to 0.8. Note that the phase difference density for PRC 2-PRC 2 pair is more peaked than that for PRC 1-PRC 1 pair, while both densities are more peaked than the heterogeneous “PRC 1-PRC 2” pair. As noted above, the peak of the heterogeneous pair is not at the origin but rather, is shifted to the left. In order to get a global measure of synchrony, we plot OP as a function of the input correlation (Figure 3C). As *c* → 1, both homogeneous pairs approach OP = 1 (i.e., perfect synchrony) while the heterogeneous pair never exceeds OP = 0.4. However, at low correlations, the heterogeneous pair can actually synchronize better than the PRC 1-PRC 1 pair (compare red to green lines in inset). That is, in the presence of low correlations, a “good synchronizer” paired with a “bad synchronizer” performs better than the homogeneous pair of bad synchronizers. This effect is not just due to our approximate expansion as the Monte Carlo simulations show the same phenomenon. Figure 3D further hints that we can also see the effect in the full ML model, although the results are not as clear.

#### 3.2.1. Experimental evidence

**Figure 4:**
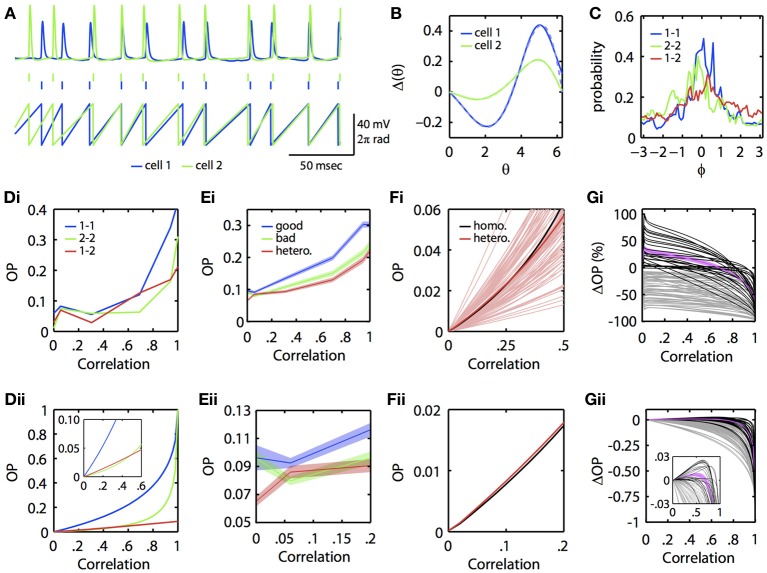
**Physiological neuronal heterogeneity can enhance correlated noise-induced synchronization at low input correlations. (A)** Example linear interpolation of phase between recorded spike times of two mitral cells injected with perfectly correlated colored noise. Top: experimentally recorded membrane potentials. Middle: raster plot of spike times. Bottom: phase. **(B)** Experimentally estimated PRCs for the two cells shown in **(A)**. Dashed lines are fits of the exponential-sine PRC model to the estimated PRCs. **(C)** Phase difference densities of the two cells during injection of perfectly correlated currents. Densities were calculated from pairs of 5 sec recordings. Blue and green curves show densities for homogeneous pairs of cell 1 and cell 2, respectively. The red curve shows the density for the heterogeneous pair of cell 1 with cell 2. **(Di)** Experimental and **(Dii)** theoretical OP vs. input correlation for homogeneous and heterogeneous pairs of the two mitral cells. Theoretical curves calculated by solving the BVP with the model PRC fits and τ = 5. [Note that the same results were obtained in separate calculations for τ = 3, the time scale of the noise used in Burton et al. (2012)]. **(Ei–Eii)** Mean OP (±SEM) vs. input correlation across 85 pairs of mitral cells (formed from 27 separate mitral cell recordings described in Burton et al. (2012)). For each pair, the cell with the greatest area under its homogeneous OP vs. correlation curve was classified as the “good synchronizer” of the pair. **(Fi)** Theoretical OP vs. input correlation (with τ = 3) for each of the 85 heterogeneous pairs from **(E)** (light red lines), plotted against the theoretical OP vs. input correlation of a homogeneous pair formed from the average mitral cell PRC. Note that many (but not all) of the heterogeneous pairs exceed the homogeneous pair in the low correlation range shown. On average (dark red line), physiological heterogeneity enhances synchrony for input correlations up to ~0.27. **(Fii)** Magnification of the homogeneous and average heterogeneous lines from **Fi** for low input correlations. **(Gi)** Percent and **(Gii)** absolute change in theoretical OP for heterogeneous vs. homogeneous bad pairs of mitral cells. Black lines plot OP changes for pairs in which heterogeneity increased synchrony at low input correlations; magenta line plots mean OP enhancement (±SEM) for these pairs. Grey lines plot OP changes for pairs in which heterogeneity did not increase synchrony. Note that heterogeneity mediates the greatest percent increase in OP at low (<0.1) input correlations, similar to experimental results shown in **(E)**. Inset shows magnification when |ΔOP| < 0.03.

Could this subtle difference in the ability of neural oscillators to transfer correlation be seen in experiments? To answer this, we re-examined data from a previous study (Burton et al., 2012). Mitral cells from the mouse main olfactory bulb were injected with constant current overlaid with frozen noise to evoke noisy periodic firing. PRCs were then experimentally estimated using our previously described method using the spike-triggered average (Ermentrout et al., 2007). [Complete methods are provided in Burton et al. (2012)]. In this dataset, we found several examples where injecting partially correlated noise produced greater synchrony between two different mitral cells firing at the same rate than for one of the mitral cells across different trials (experimentally simulating a homogeneous pair of mitral cells). Figure 4 illustrates an example. In Figure 4A, we show the voltage traces (top) of two mitral cells receiving correlated inputs, and the spike times (middle) and phase (bottom) as determined by a simple linear interpolation between spikes. Figure 4B shows the PRCs from each of these two cells along with their fit to the exponential-sine PRC model (see Appendix, Equation 64). In Figure 4C, we show the phase difference density as constructed from the linear phase interpolation of the two cells. In this example, the currents delivered through the electrodes are perfectly correlated. However, unlike the simulations, the neurons themselves are intrinsically noisy, so there is a substantial component of “private” noise. Nevertheless, one can see that cell 1 (blue) synchronizes better across trials than does cell 2 (green) across trials. Figures 4Di,Dii show the OP as reconstructed from the experimental data and as obtained by using the computed PRCs, respectively. This shows that at low correlations, the heterogeneous pair (“1–2”) can synchronize better than the “2–2” homogeneous pair (but not the “1–1” homogeneous pair). The inset in 4Dii magnifies the low *c* region.

Are the results presented in Figures 4A–D for a single pair of mitral cells consistent across a larger population of mitral cells? To answer this, we examined recordings from 27 regularly firing mitral cells, from which we were able to form 85 different pairs of mitral cells with highly similar (≤5 Hz difference) firing rates. For each pair of mitral cells, we computed the OP across varying input correlations for both homogeneous combinations and the heterogeneous combination. We automatically classified the mitral cell with the greatest homogeneous OP across all levels of input correlation as the “good synchronizer” of the mitral cell pair. Figure 4E shows the mean OP vs. correlation across the 85 good, bad, and heterogeneous mitral cell pairs. Note that, even with this relatively insensitive classification of good vs. bad synchronizers, there is a region at low input correlations where, on average, heterogeneous pairs synchronize better than the bad homogeneous pairs. This phenomenon is seen more clearly when we use the experimentally estimated PRCs and the BVP to compute the OP vs. input correlation. Figure 4Fi plots OP vs. *c* for all heterogeneous pairs (light red lines), the mean of the heterogeneous pairs (dark red line), and the OP for a single homogeneous pair whose PRC is the mean of all the PRCs (black line). For many cases (but not all), heterogeneity increases the OP above that achieved by a uniform population of neural oscillators with the mean PRC. Figure 4Fii magnifies the mean OP vs. *c* curves at low correlation; the red curve is clearly higher than the black curve.

We then quantified the degree to which physiological levels of heterogeneity [as experimentally measured in mitral cells (Burton et al., 2012)] can enhance synchrony between neural oscillators. Using the BVP and our experimentally estimated mitral cell PRCs, we calculated the percent and absolute change in OP for all 85 heterogenous vs. homogeneous bad mitral cell pairs. That is, for the example pair in Figure 4Dii, we subtracted the green from the red line to calculate the absolute change in OP, and divided this difference by the green line to calculate the percent change in OP. Figures 4Gi,Gii plot the results of this analysis for all 85 pairs. In 26 of these pairs (plotted in black), heterogeneity enhanced synchrony at low input correlations, with a mean increase in OP (plotted in magenta; ±SEM) of up to 36%. Thus, in relative terms, physiological levels of heterogeneity can significantly enhance correlated noise-induced synchrony at low input correlations. While this relative enhancement in synchrony corresponds to an admittedly low absolute increase in OP of up to 0.01 on average (Figure 4Gii), we nevertheless expect this phenomenon to significantly contribute to patterns of oscillatory synchrony in the olfactory bulb and potentially other brain regions (see Discussion).

#### 3.2.2. Good vs. bad synchronizers

When is a neuron a good vs. bad synchronizer? Here, the BVP is much simpler since we just have to compare homogeneous pairs. In this case, the probability density function can be written as:

(21) $$R(\phi )=\frac{N}{1-c\frac{g(\phi )}{g(0)}}$$

where *N* is a normalization and *g*(ϕ) is defined above by setting *n* = *m*. For low values of *c*, we get:

(22) $$R(\phi )\approx N\left[1+c\frac{g(\phi )}{g(0)}\right]$$

and integrating, we can find *N*:

(23) $$\frac{1}{N}\approx 2\pi \left[1+c\frac{1}{2\pi }\int_{0}^{2\pi }g(\phi )/g(0)\;d\phi \right]$$

Since the two neurons are identical, the peak of *R*(ϕ) occurs at ϕ = 0 and, so, we can estimate the zero lag cross-correlation as [*R*(0) − 1/(2π)]/(2π). Using the approximations above, we see that:

(24) $$\text{CC}\approx \frac{c}{2\pi }\left(1-\frac{1}{2\pi }\int_{0}^{2\pi }\frac{g(\phi )}{g(0)}\;d\phi \right):=cS$$

That is, the cross-correlation is linearly proportional to the input correlation (for small *c*) and this factor [called the susceptibility (de la Rocha et al., 2007)], is a simple function of *g*(ϕ). We can maximize *S* if we can make the integral as small as possible. Note that *g*(ϕ) is periodic, and the integral over a period is proportional to the constant Fourier coefficient. Recall that *g*(ϕ) is a low-pass filtered version of *h*(ϕ), which is the autocorrelation function of the PRC. Thus, *h*(0) is positive and so is *g*(0). The integral of *g*(ϕ) is proportional to the integral of *h*(ϕ), which is just 2π*a*^2^_0_ where *a*_0_ is the DC component of the PRC. Hence, we can minimize the integral and maximize the correlation transfer (susceptibility) if we mimimize the DC component of the PRC. This fact generalizes the conclusions in Marella and Ermentrout (2008) and Abouzeid and Ermentrout (2009) that state that more sinusoidal PRCs are the best synchronizers. For example, with a PRC of the form (sin(*a*) − sin(*x* + *a*)) *exp*(*C*(*x* − 2π)), we obtain the best synchrony when *a* = −arctan *C*.

Can we determine when a pair of oscillators will have the property that a good-bad heterogeneous pair is better than a bad-bad homogeneous pair? Since the effect is only seen at low correlations, this suggests a perturbation expansion for small *c*. We write *R*(ϕ) = ∑ *c*^*n*^ *R*_*n*_(ϕ) and find that *R*_0_ is constant and so to order 1, *R*(ϕ) = *R*_0_ + *cR*_1_(ϕ). From this, we can compute OP, *OP* = *c*∫^2π^_0_ cosϕ *R*_1_(ϕ) *d*ϕ. The formulas for this are not terribly useful, but we can illustrate the results with a simple example. Let Δ_*j*_(ϕ) = sin(*a*_*j*_) − sin(ϕ + *a*_*j*_), where *j* = 1, 2 for two oscillators. Then:

(25) $${\text{OP}}_{jk}=c\frac{K}{1+\left(\tau^{2}+1\right)\left[\sin^{2}a_{j}+\sin^{2}a_{k}\right]}$$

Thus, for 0 ≤ *a*_1_ < *a*_2_ ≤ π/2, we always have *OP*_11_ > *OP*_12_ > *OP*_22_ for all τ and sufficiently small values of *c*. This provides a simple and surprising illustration that heterogeneity will improve synchrony at low correlations for very simple PRCs. We remark, however, that this phenomenon does not always hold. Pairs of PRCs can be found such that OP is always bigger for both of the homogeneous oscillator pairs than for the heterogeneous oscillator pair, as can be seen from Figures 4F,G.

#### 3.2.3. PRC heterogeneity tunes the sharpness and peak position of the phase difference density

**Figure 5:**
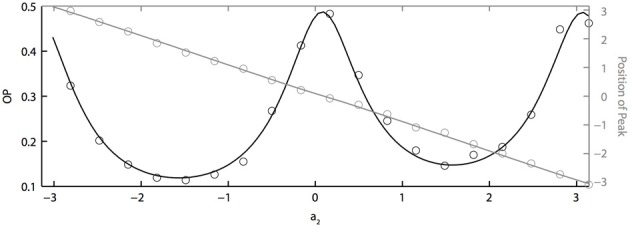
**PRC heterogeneity tunes the sharpness and peak position of the phase difference density.** OP vs. *a*_2_ (black axis) and the peak position of the phase difference density vs. *a*_2_ (grey axis). Parameters used: *a*_1_ = 0.1, *b*_1_ = 0.32, *b*_2_ = 0.3, τ = 1, and *c* = 0.8.

If two neurons are identical but driven with partially correlated noise, then the peak of the phase difference density will be centered at ϕ = 0, which means that the two oscillators will tend to have the same phase. However, with heterogeneity, the peak will shift depending on the degree of heterogeneity, just as two coupled oscillators will shift if they have different intrinsic frequencies. Figure 5 shows how the peak of the phase difference density is shifted by oscillator heterogeneity. Using the two term double sinusoidal form PRC (Equation 63), we keep PRC 1 constant (*a*_1_ = 0.1, *b*_1_ = 0.32) as we vary PRC 2 (*b*_2_ = 0.3 is constant and *a*_2_ varies from −π to π). From the results shown in Figure 5, we can conclude that heterogeneity can tune oscillator synchronization in both the sharpness and peak position of the phase difference density, which might be useful in neural signal coding. We also note that OP is minimized when the peak is at ± π/2 and that “changing the sign” of the PRC (e.g., setting *a*_2_ = π) shifts the peak but has very little effect on the OP.

### 3.3. Frequency differences highly limit synchronization

**Figure 6:**
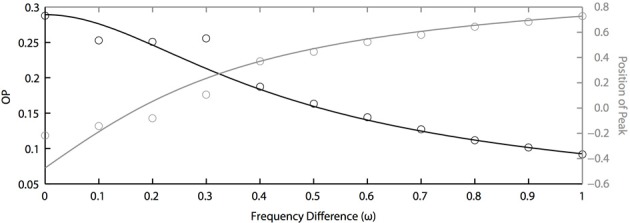
**Frequency differences limit noise-induced synchronization.** OP decreases quickly as frequency differences increase (black axis). The peak position of the phase difference density is shifted by changing frequency differences between two oscillators (grey axis). Parameters used here: *a*_1_ = 0.1, *b*_1_ = 0.32, *a*_2_ = 0.6, *b*_2_ = 0.3, τ = 1, and *c* = 0.8.

In the above results, we assume that all oscillators have the same natural frequency, which means ω = 0. This is a somewhat unreasonable assumption for real neurons. Thus, we now study how synchronization is dependent on the frequency differences between oscillators. Figure 6 shows the effects of frequency differences on a pair of oscillators that have different PRCs (of the two term double sinusoidal form, Equation 63) and are driven by partially correlated noise. With no frequency difference, the heterogeneity in oscillator PRCs yields a shift in the peak position (Figure 5), consistent with previous measurements of synchrony between irregularly firing neurons (Tchumatchenko et al., 2010). This means that, if frequency differences can shift the peak in the opposite direction [e.g., see Figure 1C of Burton et al. (2012)], then changes in frequency could “cancel” the effects of PRC heterogeneity so that the peak of the phase difference density is at 0. This cancellation can be seen in Figure 6 near ω = 0.2. However, this cancellation comes at a loss to precision, as seen by the decrease in OP. While not shown, we remark that the drop in OP is symmetric about ω = 0; thus, a negative frequency difference will not result in a larger OP. While it remains to be proven, we conjecture that the OP is always maximal when there is no frequency difference. This differs from the case that we looked at in the previous section where heterogeneity (in PRCs) can sometimes lead to a larger OP than homogeneity.

### 3.4. Correlated noise-induced synchronization is dependent on the time constant of the noise

**Figure 7:**
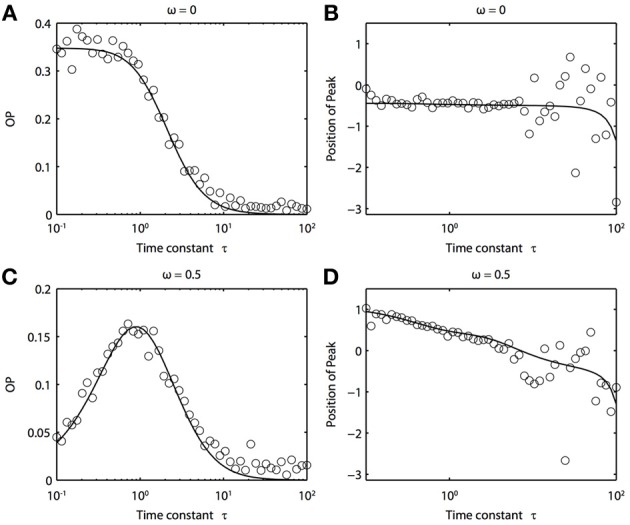
**Frequency differences between oscillators change the dependence of synchrony on the time constant of the correlated noise. (A)** OP and **(B)** the peak position of the phase difference density for oscillators with no frequency difference (ω = 0). **(C)** OP and **(D)** the peak position of the phase difference density for oscillators with a frequency difference (ω = 0.5). Parameters used: *a*_1_ = 0.1, *b*_1_ = 0.32, *a*_2_ = 0.6, *b*_2_ = 0.3, and *c* = 0.8.

**Figure 8:**
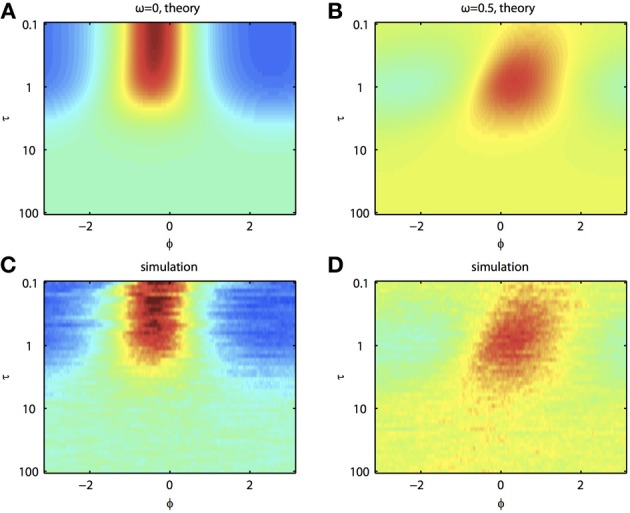
**Synchronization of oscillators with different frequencies resonates with the time constant of the correlated noise. (A)** BVP solution and **(C)** results of Monte Carlo simulation for *R*(ϕ) between two oscillators with no frequency difference (ω = 0) as τ varies. X-axis shows the phase difference, ϕ, and higher *R*(ϕ) is plotted as hotter colors in the heat map. **(B)** and **(D)**, same as **(A)** and **(C)** for two oscillators with a frequency difference (ω = 0.5). Parameters used: *a*_1_ = 0.1, *b*_1_ = 0.32, *a*_2_ = 0.6, *b*_2_ = 0.3, and *c* = 0.8.

**Figure 9:**
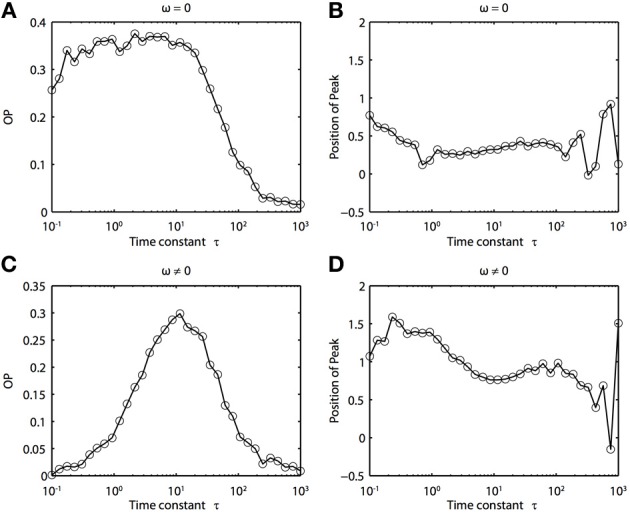
**Frequency differences between biophysically realistic spiking neuron models change the dependence of synchrony on the time constant of the correlated noise. (A)** OP and **(B)** peak position for model neurons with different PRCs but the same frequency (*I*_1_ = 120, ϕ_1_ = 0.04, *I*_2_ = 110, and ϕ_2_ = 0.04616). **(C)** OP and **(D)** peak position for model neurons with different PRCs (same as above) and slightly different frequencies (*I*_1_ = 120, ϕ_1_ = 0.041, *I*_2_ = 110, and ϕ_2_ = 0.04616).

Because of the natural decay times of synapses, broadband inputs into neurons have some temporal correlations. Thus, we now explore how the temporal properties of noise interact with heterogeneities in the PRCs. Figure 7 shows that synchronization decreases monotonically as τ increases for ω = 0, while there exists an optimal value of τ that achieves the greatest synchronization for ω = 0.5. This means synchronization of two oscillators with different frequencies (i.e., ω ≠ 0) can have a resonance with τ. Furthermore, as seen in Figures 7B,D the peak of the phase difference density depends on τ only when there is a frequency difference between the two oscillators. In Figure 8, we explore this resonance in more detail where *R*(ϕ) is plotted as τ varies. The left panels (showing the solution to the BVP and the results of Monte Carlo simulation) show that when ω = 0, the peak position of *R*(ϕ) is largely unchanged and the magnitude decreases monotonically with τ. There is a sharp drop off in *R*(ϕ) at τ ≈ 2. A different result emerges in the right panels, where a frequency difference exists (ω = 0.5). At low and high values of τ, *R*(ϕ) is almost flat with a distinct resonance when τ ≈ 1. We see the same resonance in the biophysical ML model when the neurons have different frequencies and different PRCs (Figure 9).

We can see why the frequency differences are needed for resonance by considering the simplest example of identical PRCs of the form Δ(ϕ) = sin *a* − sin(ϕ + *a*). In this case, we solve the BVP:

(26) $$\frac{G(\phi ,\tau )R(\phi )}{d\phi }=\alpha \frac{1+\tau^{2}}{\tau }(R(\phi )-1)$$

where *G*(ϕ, τ) = (1 + τ^2^) sin(*a*)^2^ + 1 − *c* cosϕ. Here, α is proportional to the frequency difference. In particular, note that when ω = 0, *G* is independent of τ and otherwise, τ acts to weaken the correlated noise-induced synchronization as it increases the part of *G* that is not phase dependent. However, the right side of this equation shows that the effect of the frequency difference is minimized when τ = 1, and thus we expect resonance in the OP. This effect disappears when α = 0.

## 4. Discussion

In our current study, we have extended a number of previous results describing the ability of neural oscillators to synchronize in the presence of correlated noise. Our methods are similar to those in Burton et al. (2012), with the additional aspect that we now use colored noise (OU process). The properties of the noise show up only through a convolution of the autocorrelation function of the noise with the phase functions *h*_*nm*_(ϕ) that, in turn, depend only on the PRCs (see Appendix, Equation 56). Thus, we could easily generalize this work to noise with an arbitrary autocorrelation function. In addition, we have now included many more examples of the theory and shown that the conclusions from the perturbation theory continue to be valid for full biophysical models (cf. Figure 2). Further, we have shown that for low input correlations, heterogeneity can actually improve synchrony both pairwise and in large populations. We demonstrated that this theoretical effect can be seen in experimental recordings of regularly firing olfactory bulb mitral cells. Thus, we have significantly extended the findings presented in Burton et al. (2012), and our results on colored noise further suggest some experimentally testable phenomena, such as the resonance seen in slightly detuned oscillators (Figures 7–9). These novel findings and their biological implications are discussed in more detail below.

### 4.1. Heterogeneity can improve synchrony

We found that correlated noise can synchronize a heterogeneous pair of oscillators (comprised of a “bad synchronizer” and a “good synchronizer”) better than a homogeneous pair of bad synchronizers at low levels of input correlation and verified this experimentally. We showed that good (bad) synchronizers are characterized by having a relatively low (high) DC component in their PRC. Consistent with this, oscillators with the generic “type II” PRC (i.e., sin ϕ) are better synchronizers than oscillators with the generic “type I” PRC (i.e., 1 − cosϕ).

Several authors have previously studied the effects of heterogeneity on the transfer of correlation. As we noted in Introduction, at low correlations, the output correlation is linearly proportional to the input correlation through a factor, *S*, called the susceptibility (de la Rocha et al., 2007; Shea-Brown et al., 2008). If we let *S(A, B)* denote the susceptibility for two neurons, *A, B*, then what we have found in our current study is that in some cases, *S*(*A*, *A*) > *S*(*A*, *B*) > *S*(*B*, *B*). Note that in our study, we are looking at output correlation related to spike-to-spike synchronization, whereas in many other studies of output correlation, the interest is in *spike count* correlation. We can regard our measure of synchrony as the same as spike count correlation, but over a time window that is of the order of the mean interspike interval. In a recent paper, (Shea-Brown et al., 2008) showed that for spike count correlation, $S(A,\;B)=\sqrt{S(A,\;A)S(B,\;B)}$ and thus, trivially, we can obtain *S*(*A*, *A*) > *S*(*A*, *B*) > *S*(*B*, *B*) when *A* is “better” than *B* at transferring correlation. We want to emphasize that their result is for long time windows (that is, the window length tends to infinity). Which neurons are better than others at the transfer of correlation depends very strongly on the window of time through which you measure the correlation. Indeed, Barreiro et al. (2010) and Abouzeid and Ermentrout (2011) showed that type II PRCs have larger susceptibilities than type I for short time windows (i.e., spike synchrony) but the trend is reversed for large time windows (i.e., rate correlation).

Interestingly, the efficiency of correlated-noise induced synchronization is also modulated by firing rate in the low input correlation regime (de la Rocha et al., 2007; Tchumatchenko et al., 2010). Given that changes in firing rate can modulate PRC shape in biophysically realistic neuron models and in real neurons (Gutkin et al., 2005; Marella and Ermentrout, 2008; Stiefel et al., 2008, 2009; Schultheiss et al., 2010; Fink et al., 2011; Burton et al., 2012), whether or not (and the degree to which) PRC heterogeneity will enhance synchrony may depend in part on the firing rate. However, in the simplest cases (such as models like the leaky-integrate and fire model and the theta model), the only effect of the firing rate on the shape of the PRC is to change its amplitude. Since amplitude (but not shape) changes can be absorbed into the size of the noise, and our theory shows that the phase difference density is independent of the size of the noise (at least, if it is small enough), changes in firing rate will have no effect on the synchronization of pairs of neurons firing at the same or nearly the same rates.

The ability of cellular heterogeneity to regulate which oscillators synchronize best as a function of input correlation likely contributes to coding in many neural systems. In the olfactory bulb, where oscillatory synchrony appears to be critical to olfactory coding [for review, see Bathellier et al. (2010)], tens of “sister” mitral cells are linked to each glomerulus where they receive highly correlated afferent input (Carlson et al., 2000; Schoppa and Westbrook, 2001). Each sister mitral cell of a glomerulus may also participate in independent (i.e., unshared) lateral inhibitory circuits with non-sister mitral cells of surrounding glomeruli, mediated by local inhibitory granule cells (Dhawale et al., 2010; Tan et al., 2010). On average, sister mitral cells are thus subject to high input correlations while non-sister mitral cells are subject to low (though non-zero) input correlations (Dhawale et al., 2010). Further, we and others have demonstrated that mitral cells exhibit substantial cell-to-cell heterogeneity (Padmanabhan and Urban, 2010; Angelo and Margrie, 2011; Angelo et al., 2012; Burton et al., 2012). Based on our current results, this heterogeneity will thus act to reduce output synchrony of sister mitral cells but *enhance* output synchrony of non-sister mitral cells. Thus, in the context of the olfactory system, heterogeneity will promote encoding of combinatorial sensory information (i.e., activation of non-sister mitral cells by odor combinations).

Our results suggest that heterogeneity can only enhance correlation-induced synchronization by a moderate amount between two neural oscillators (up to 36% in BVP solutions using mitral cell PRCs). Two properties of the olfactory bulb nevertheless suggest that even this moderate effect can significantly influence patterns of oscillatory synchrony in the olfactory system. First, the reciprocal dendrodendritic connectivity between mitral cells and granule cells enables activity-dependent regulation of granule cell recruitment (Arevian et al., 2008), which can lead to amplification of granule cell-mediated correlated noise-induced synchronization (Marella and Ermentrout, 2010). Second, mitral cells separated by up to ~2 mm can engage in lateral inhibitory interactions (Egger and Urban, 2006), thus multiplying the synchrony-enhancing effect of cellular heterogeneity across a potentially large fraction of the ~40,000 total mitral cells per mouse olfactory bulb (Benson et al., 1984). Whether neural oscillator heterogeneity exists in, and significantly enhances, correlated-noise induced synchrony in other brain regions remains a promising topic of future research.

### 4.2. Resonance

In addition to the above findings, we found that there exists some resonance of correlated noise-induced synchronization with respect to the time scale of the noise. That is, we found a local maximum in OP as the time scale of the correlated noise varied. Surprisingly, this only occurs when there is a difference in the frequencies between the two oscillators. The requirement for the frequency difference would seem to contradict earlier work (Galán et al., 2008), where it was found that the Liapunov exponent was most negative when the noise has a particular time scale. However, when the noise is only partially correlated, the uncorrelated part of the noise causes a drift in the phase difference. The degree of this drift is also dependent on the time scale of the noise, and thus the two effects cancel. A frequency difference breaks this symmetry by adding an additional drift term, which prevents one from factoring out the resonance. A frequency difference thus leads to a dependence of OP on the time scale of the noise. We have not yet tested this idea experimentally, but it seems to be quite robust, having been found in both the simple phase models (Figures 7, 8) and in the biophysical ML model (Figure 9).

### 4.3. Limitations of the theory

The analysis that we have done in this paper and in our earlier papers requires that the neurons fire almost periodically. This means that the activity of the neurons is *mean driven* rather than *fluctuation driven* so that the coefficient of variation of the interspike intervals should be small. While this may not be the case in all areas of the brain, there are many regions, such as the olfactory bulb, where the firing rate can be quite regular and synchronous as indicated by the large rhythmic local field potentials. Assuming that the neurons are firing at a fairly regular rate, it is also reasonable to ask how well the PRC describes such noisy neurons. An extensive review of the caveats of PRC theory for real neurons can be found in Smeal et al. (2010). Another issue is the actual estimation of the PRCs in the presence of noise. Several studies have shown that background synaptic activity and other forms of noise do not significantly affect the shape of the PRC (Ermentrout et al., 2011; Netoff et al., 2012).

In conclusion, we have extended our previous work to demonstrate that oscillator heterogeneity and frequency differences interact with the time scale of input noise to regulate how correlated noise synchronizes uncoupled oscillators.

### 4.4. Data sharing

All codes are available by request from the authors. They include Matlab and XPPaut files.

### Conflict of interest statement

The authors declare that the research was conducted in the absence of any commercial or financial relationships that could be construed as a potential conflict of interest.

## Appendix

### Reduction to a phase model

Consider a general oscillator receiving a possibly noisy time-dependent signal:

(27) $$\frac{dX}{dt}=F(X)+\epsilon N(X,t)$$

Here *N*(*X*, *t*) is the external or imposed inputs into the system. For single compartment neural models, *N* will typically only affect the membrane potential, e.g., as an injected or synaptic current. We assume that *X*′ = *F*(*X*) has as a solution an exponentially stable limit cycle, *U*(*t* + *T*) = *U*(*t*) and that ϵ is a small positive parameter characterizing the magnitude of the input. We are interested in how the phase of the limit cycle evolves in time in the presence of small inputs. The phase of a limit cycle is easy to define when a point lies *on* the limit cycle. For example, for neurons, the phase is just the time since the last spike of the cell. However, if the limit cycle is attracting, then it is also possible to define the phase of a point that is near, but not directly on the limit cycle. Specifically, there is a function Θ(*X*) that maps a point near the limit cycle, *X*, to the phase that it will eventually reach as it is attracted to the limit cycle (asymptotic phase). Clearly Θ(*U*(*t*)) = *t*. Define the phase to be θ(*t*) = Θ(*X*(*t*)), so that by the chain rule:

(28) $$\frac{d\theta }{dt}=\nabla_{X}\Theta (X)\cdot \frac{dX}{dt}$$

(29) $$=\nabla_{X}\Theta (X)\cdot F(X)+\epsilon \nabla_{X}\Theta (X)\cdot N(X(t),t)$$

(30) $$=1+\epsilon \nabla_{X}\Theta (X)\cdot N(X(t),t)$$

Thus, in the absence of inputs, the phase moves around the circle at constant velocity. This expression is exact, but not very helpful since it requires knowledge of the solution *X*(*t*). Kuramoto's approximation (which is valid for small ϵ) is to replace *X*(*t*) in the right-hand side by *U*(θ(*t*)), where *U* is the unperturbed limit cycle (Kuramoto, 2003). This closes the system yielding:

(31) $$\frac{d\theta }{dt}=1+\epsilon Z(\theta )\cdot N(U(\theta ),t)$$

where we have defined *Z*(θ): = ∇_*X*_Θ(*U*(θ)). The function, *Z*(θ) is the so-called adjoint function satisfying the linear equation:

(32) $$Z^{\prime }=-{\left(D_{X}F(U(t))\right)}^{T}Z(t)$$

with *Z*(*t*) · *U*′(*t*) = 1. Here *D*_*X*_*F*(*U*(*t*)) means the linearization of *F*(*X*) evaluated along the limit cycle.

In single compartment neuron models, inputs appear only in the voltage component of the neural oscillator in the form of external currents so that the dot product in Equation 31 becomes scalar multiplication:

(33) $$\frac{d\theta }{dt}=1+\epsilon \Delta (\theta )I(U(\theta ),t)/C$$

where *I* is the input current, *C* is the capacitance, and Δ(θ) is the voltage component of the vector *Z*. The quantity, Δ(θ), is sometimes called the infinitesimal PRC and, for small perturbations, is proportional to the PRC.

### Derivation of the stationary density of phase differences

The Langevin equations that drive the phase models (Equations 4–6) correspond to a forward Fokker-Planck (FP) equation that can be written as (Risken, 1984):

(34) $$\begin{array}{l} \frac{\partial \rho }{\partial t}=\frac{1}{\tau }\left\{\frac{\partial }{\partial x}\left(\frac{1}{2}\frac{\partial }{\partial x}+x\right)+\frac{\partial }{\partial y}\left(\frac{1}{2}\frac{\partial }{\partial y}+y\right)+c\frac{\partial^{2}}{\partial x\partial y}\right\}\rho -\frac{\partial }{\partial \theta }\rho \\ \;-\epsilon \left\{\frac{\partial }{\partial \theta }[\Delta_{1}(\theta )x\rho ]+\frac{\partial }{\partial \phi }[(\Delta_{2}(\theta +\phi )y-\Delta_{1}(\theta )x)\rho ]\right\}\\ \;-\epsilon^{2}\omega \frac{\partial }{\partial \phi }\rho \end{array}$$

When the distribution of phase differences is stationary, $\frac{\partial \rho }{\partial t}=0$. Our goal is to exploit the smallness of ϵ to compute this stationary density with which we can compute the marginal distribution of the phase difference.

### Analytical solution

We expand the steady state ρ in ϵ:

(35) $$\begin{array}{l} \rho (x,y,\theta ,\phi )=\rho_{0}(x,y,\theta ,\phi )+\epsilon \rho_{1}(x,y,\theta ,\phi )+\epsilon^{2}\rho_{2}(x,y,\theta ,\phi )\\ \int \text{​}\int \text{​}\int \text{​}\int \rho_{0}(x,y,\theta ,\phi )dxdyd\theta d\phi =1\\ \int \text{​}\int \text{​}\int \text{​}\int \rho_{n}(x,y,\theta ,\phi )dxdyd\theta d\phi =0,\;n=1,2 \end{array}$$

We define the operator:

(36) $$L_{0}=\frac{1}{\tau }\left\{\frac{\partial }{\partial x}\left(\frac{1}{2}\frac{\partial }{\partial x}+x\right)+\frac{\partial }{\partial y}\left(\frac{1}{2}\frac{\partial }{\partial y}+y\right)+c\frac{\partial^{2}}{\partial x\partial y}\right\}+\frac{\partial }{\partial \theta }$$

At steady state condition ($\frac{\partial \rho }{\partial t}=0$), we substitute the above expansion into the FP equation and collect the powers of ϵ. We need to go to ϵ^2^:

(37) $$0=L_{0}\rho_{0}$$

(38) $$0=L_{0}\rho_{1}-\left\{\frac{\partial }{\partial \theta }[\Delta_{1}(\theta )x\rho_{0}]+\frac{\partial }{\partial \phi }[(\Delta_{2}(\theta +\phi )y-\Delta_{1}(\theta )x)\rho_{0}]\right\}$$

(39) $$0=L_{0}\rho_{2}-\left\{\frac{\partial }{\partial \theta }[\Delta_{1}(\theta )x\rho_{1}]+\frac{\partial }{\partial \phi }[(\Delta_{2}(\theta +\phi )y-\Delta_{1}(\theta )x)\rho_{1}]\right\}-a\frac{\partial }{\partial \phi }\rho_{0}$$

#### Solving equation 37

Equation 37 is just a linear separable equation, independent of ϕ, so, by inspection:

(40) $$\rho_{0}(x,y,\theta ,\phi )=\frac{1}{2\pi }G(x,y)R(\phi )$$

where:

(41) $$G(x,y)=\frac{1}{\sqrt{1-c^{2}}\pi }e^{-\frac{1}{1-c^{2}}(x^{2}+y^{2}-2cxy)}$$

and *R*(ϕ) remains to be determined. Note that *G*(*x*, *y*) is just the standard stationary solution to the multivariate OU equation. At this juncture, we remark that our main goal is to find *R*(ϕ), which is the marginal density of the phase differences between the two oscillators.

#### Solving equation 38

Both Equations 38 and 39 have the form *L*_0_ρ = *b*(*x*, *y*, θ). *L*_0_ operates on the space of functions defined on *R*^2^× *S*^1^ that are twice continuously differentiable in *x, y* and continuously differentiable in θ. In this space, *L*_0_ has a one-dimensional nullspace spanned by *G*(*x*, *y*) (constant in θ) and so *L*_0_ is not invertible. However, *L*_0_ρ(*x*, *y*, θ) = *b*(*x*, *y*, θ) does have a solution provided that *b*(*x*, *y*, θ) is orthogonal to the null space of *L*^*^_0_, which is the adjoint linear operator of *L*_0_. Since *L*_0_ is a standard probability operator, its adjoint is always 1 (i.e., the function that is 1 everywhere).

Since:

(42) $$b_{1}(x,y,\theta )=\frac{xG(x,y)}{2\pi }[\Delta_{1}^{\prime }(\theta )R(\phi )-\Delta_{1}(\theta )R^{\prime }(\phi )]+yG(x,y)[\Delta_{2}^{\prime }(\theta +\phi )R(\phi )+\Delta_{2}(\theta +\phi )R^{\prime }(\phi )]$$

we see that ∭ *b*_1_(*x*, *y*, θ)*dxdyd*θ = 0. Thus, *L*_0_ρ_1_ = *b*_1_ has a solution. Since:

(43) $$L_{0}[xG(x,y)]=-xG(x,y)/\tau$$

(44) $$L_{0}[yG(x,y)]=-yG(x,y)/\tau$$

we look for a solution of the form:

(45) $$\rho_{1}(x,y,\theta ,\phi )=\frac{w_{1}(\theta ,\phi )xG(x,y)+w_{2}(\theta ,\phi )yG(x,y)}{2\pi }$$

Inserting this into Equation 38, we find that *w*_*j*_(θ, ϕ) must satisfy:

(46) $$\frac{\partial }{\partial \theta }w_{1}(\theta ,\phi )+\frac{w_{1}(\theta ,\phi )}{\tau }=-\Delta_{1}^{\prime }(\theta )R(\phi )+\Delta_{1}(\theta )R^{\prime }(\phi )$$

(47) $$\frac{\partial }{\partial \theta }w_{2}(\theta ,\phi )+\frac{w_{2}(\theta ,\phi )}{\tau }=-\Delta_{2}^{\prime }(\theta +\phi )R(\phi )-\Delta_{2}(\theta +\phi )R^{\prime }(\phi )$$

*w*_*j*_ must be periodic functions of θ; we defer their exact solution to later, but note that there is always a unique periodic solution to each of these equations.

#### Solving equation 39

We now have:

(48) $$b_{2}(x,y,\theta )=\frac{\partial }{\partial \theta }[\Delta_{1}(\theta )x\rho_{1}]+\frac{\partial }{\partial \phi }[(\Delta_{2}(\theta +\phi )y-\Delta_{1}(\theta )x)\rho_{1}]+a\frac{\partial }{\partial \phi }\rho_{0}$$

In order to solve Equation 39, for ρ_2_(*x*, *y*, θ, ϕ), we must have:

(49) $$\begin{array}{l} 0=\int \text{​}\text{​​}\int_{-\infty }^{\infty }\int_{0}^{2\pi }b_{2}(x,y,\theta )dxdyd\theta \\ \;=0+\frac{\partial }{\partial \phi }\{\int \text{​}\text{​​}\int_{-\infty }^{\infty }\int_{0}^{2\pi }\{\frac{\Delta_{2}(\theta +\phi )}{2\pi }[w_{2}(\theta ,\phi )y^{2}+w_{1}(\theta ,\phi )xy]G(x,y)\\ \;-\frac{\Delta_{1}(\theta )}{2\pi }[w_{1}(\theta ,\phi )x^{2}+w_{2}(\theta ,\phi )xy]G(x,y)+\frac{a}{2\pi }R(\phi )G(x,y)\}dxdyd\theta \}\\ \;=\frac{1}{4\pi }\frac{\partial }{\partial \phi }\left\{\int_{0}^{2\pi }\{\Delta_{2}(\theta +\phi )[w_{2}(\theta ,\phi )+c\cdot w_{1}(\theta ,\phi )]-\Delta_{1}(\theta )[w_{1}(\theta ,\phi )+c\cdot w_{2}(\theta ,\phi )]\}d\theta +4\pi \omega R(\phi )\right\}\\ \;=\frac{1}{4\pi }\frac{\partial }{\partial \phi }[f(\phi )+4\pi aR(\phi )] \end{array}$$

where:

(50) $$\begin{array}{l} \;f(\phi )=\int_{0}^{2\pi }[\Delta_{2}(\theta +\phi )v_{2}(\theta ,\phi )-\Delta_{1}(\theta )v_{1}(\theta ,\phi )]d\theta \\ v_{1}(\theta ,\phi )=w_{1}(\theta ,\phi )+cw_{2}(\theta ,\phi )\\ v_{2}(\theta ,\phi )=w_{2}(\theta ,\phi )+cw_{1}(\theta ,\phi ) \end{array}$$

Given Equations 46 and 47, we see that *v*_1_(θ) and *v*_2_(θ) satisfy:

(51) $$\begin{array}{l} v_{1}^{\prime }(\theta )+\frac{v_{1}(\theta )}{\tau }=-[\Delta_{1}^{\prime }(\theta )+c\cdot \Delta_{2}^{\prime }(\theta +\phi )]R(\phi )+[\Delta_{1}(\theta )-c\cdot \Delta_{2}(\theta +\phi )]R^{\prime }(\phi )\\ \;:=q_{1}(\theta ) \end{array}$$

(52) $$\begin{array}{l} v_{2}^{\prime }(\theta )+\frac{v_{2}(\theta )}{\tau }=-[c\cdot \Delta_{1}^{\prime }(\theta )+\Delta_{2}^{\prime }(\theta +\phi )]R(\phi )+[c\cdot \Delta_{1}(\theta )-\Delta_{2}(\theta +\phi )]R^{\prime }(\phi )\\ \;:=q_{2}(\theta ) \end{array}$$

For Equations 51 and 52, we can write down the solution of *v*_1_(θ) and *v*_2_(θ) in terms of *q*_1_(θ) and *q*_2_(θ) (see Appendix):

(53) $$v_{n}(\theta )=\int_{0}^{\infty }e^{-\frac{s}{\tau }}q_{n}(\theta -s)ds,\;n=1,2$$

We substitute *v*_*n*_(ϕ) into *f*(ϕ),

(54) $$\begin{array}{l} f(\phi )=\int_{0}^{2\pi }[\Delta_{2}(\theta +\phi )v_{2}(\theta )-\Delta_{1}(\theta )v_{1}(\theta )]d\theta \\ \;=\int_{0}^{\infty }e^{-\frac{s}{\tau }}ds\int_{0}^{2\pi }[\Delta_{2}(\theta +\phi )q_{2}(\theta -s)-\Delta_{1}(\theta )q_{1}(\theta -s)]d\theta \\ \;=\int_{0}^{\infty }e^{-\frac{s}{\tau }}ds\int_{0}^{2\pi }h(\theta ,\phi ,s)d\theta \end{array}$$

where:

(55) $$\begin{array}{l} h(\theta ,\phi ,s)=\Delta_{2}(\theta +\phi )q_{2}(\theta -s)-\Delta_{1}(\theta )q_{1}(\theta -s)\\ \;=\Delta_{1}(\theta -s)[c\cdot \Delta_{2}(\theta +\phi )-\Delta_{1}(\theta )]R^{\prime }(\phi )\\ \;+\Delta_{2}(\theta +\phi -s)[-\Delta_{2}(\theta +\phi )+c\cdot \Delta_{1}(\theta )]R^{\prime }(\phi )\\ \;+\Delta_{1}^{\prime }(\theta -s)[-c\cdot \Delta_{2}(\theta +\phi )+\Delta_{1}(\theta )]R(\phi )\\ \;+\Delta_{2}^{\prime }(\theta +\phi -s)[-\Delta_{2}(\theta +\phi )+c\cdot \Delta_{1}(\theta )]R(\phi ) \end{array}$$

Define:

(56) $$\begin{array}{l} g_{mn}(\phi )=\int_{0}^{\infty }h_{mn}(s+\phi )e^{-\frac{s}{\tau }}ds\\ h_{mn}(s)=\int_{0}^{2\pi }\Delta_{m}(\theta )\Delta_{n}(\theta +s)d\theta \end{array}$$

Since Δ_1_(θ) and Δ_2_(θ) are periodic functions,

(57) $$\begin{array}{l} \int_{0}^{2\pi }h(\theta ,\phi ,s)d\theta =\int_{0}^{2\pi }\{\Delta_{1}(\theta -s)\{[c\Delta_{2}(\theta +\phi )-\Delta_{1}(\theta )]R^{\prime }(\phi )+[c\Delta_{2}^{\prime }(\theta +\phi )-\Delta_{1}^{\prime }(\theta )]R(\phi )\}\\ \;+\Delta_{2}(\theta +\phi -s)\{[c\Delta_{1}(\theta )-\Delta_{2}(\theta +\phi )]R^{\prime }(\phi )-[c\Delta_{1}^{\prime }(\theta )-\Delta_{2}^{\prime }(\theta +\phi )]R(\phi )\}\}d\theta \\ \;=\{c[h_{12}(s+\phi )+h_{21}(s-\phi )]-[h_{11}(s)+h_{22}(s)]\}R^{\prime }(\phi )\\ \;+\left\{c\frac{d}{d\phi }[h_{12}(s+\phi )+h_{21}(s-\phi )]-\frac{d}{d\phi }[h_{11}(s+\phi )-h_{22}(s+\phi )]{|}_{\phi =0}\right\}R(\phi ) \end{array}$$

(58) $$\begin{array}{l} f(\phi )=\int_{0}^{\infty }e^{-\frac{s}{\tau }}ds\int_{0}^{2\pi }h(\theta ,\phi ,s)d\theta \\ \;=\{c[g_{12}(\phi )+g_{21}(-\phi )]-[g_{11}(0)+g_{22}(0)]\}R^{\prime }(\phi )\\ \;+\left\{c\cdot \frac{d}{d\phi }[g_{12}(\phi )+g_{21}(-\phi )]-[g_{11}^{\prime }(0)-g_{22}^{\prime }(0)]\right\}R(\phi )\\ \;=\frac{d}{d\phi }\{[c\cdot g(\phi )-C_{1}]R(\phi )\}-C_{2}R(\phi ) \end{array}$$

where:

(59) $$g(\phi )=g_{12}(\phi )+g_{21}(-\phi )$$

(60) $$C_{1}=g_{11}(0)+g_{22}(0)$$

(61) $$C_{2}=g_{11}^{\prime }(0)-g_{22}^{\prime }(0)$$

Combined with Equations 49–58, we have a boundary value problem (BVP):

(62) $$\begin{array}{l} \frac{d}{d\phi }\{[c\cdot g(\phi )-C_{1}]R(\phi )\}+(4\pi \omega -C_{2})R(\phi )=K\\ \;R(\phi )=R(\phi +2\pi )\\ \;g(\phi )=g(\phi +2\pi )\\ \;\int_{-\pi }^{\pi }R(\phi )d\phi =1\\ \;K=2\omega -\frac{C_{2}}{2\pi } \end{array}$$

To solve this BVP, we need to compute *g*(ϕ) for a given PRC. We use two forms of the PRC in this paper:

(63) Δ(θ)=sin(a)−sin(θ+a)+bsin(2θ)

and

(64) $$\Delta (\theta )=A[\sin(B)-\sin(\theta +B)]e^{C(\theta -2\pi )}\;(\theta \in (0,2\pi ),\Delta (\theta )=\Delta (\theta +2\pi ))$$

The required integrals can be computed for both PRC forms. More generally, all PRCs can be written in Fourier form and, again, the integrals are readily computed to obtain *g*(ϕ) (see below).

#### Small correlation approximation for *R*(ϕ)

We use a BVP solver to get the numerical solution for *R*(ϕ), but we would like to better understand the form of *R*(ϕ) at low values of correlation, *c*, so we expand *R* as a series in *c*. As *K* is dependent on *c*, we must also expand *K* in *c*. Finally, we need to keep the normalization condition for *R*(ϕ), hence:

(65) $$\begin{array}{l} \;R(\phi )=R_{0}(\phi )+cR_{1}(\phi )+\ldots \;K=K_{0}+cK_{1}+\ldots \\ \;R_{0}(\phi )=R(\phi ){|}_{c=0},\;\int_{-\pi }^{\pi }R_{0}(\phi )d\phi =1\\ \int_{-\pi }^{\pi }R_{n}(\phi )=0,n\geq 1 \end{array}$$

We substitute these expressions into the BVP, Equation 62 and find after equating powers of *c*:

(66) $$-C_{1}R_{0}^{\prime }(\phi )+(4\pi \omega -C_{2})R_{0}(\phi )=K_{0}$$

(67) $$-C_{1}R_{1}^{\prime }(\phi )+(4\pi a-C_{2})R_{1}(\phi )+{\left[g(\phi )R_{0}(\phi )\right]}^{\prime }=K_{1}$$

We can integrate both left and right side over [−π, π], then use the assumptions and periodicity requirements above to get:

(68) $$K_{0}=\frac{4\pi \omega -C_{2}}{2\pi },\;K_{1}=0$$

Rewriting these equations,

(69) $$\begin{array}{l} R_{0}^{\prime }(\phi )+DR_{0}(\phi )=Q_{0}\\ \;D=\frac{C_{2}-4\pi \omega }{C_{1}},\;Q_{0}=-\frac{K_{0}}{C_{1}} \end{array}$$

(70) $$\begin{array}{l} R_{1}^{\prime }(\phi )+DR_{1}(\phi )=Q_{1}(\phi )\\ \;Q_{1}(\phi )=\frac{{\left[g(\phi )R_{0}(\phi )\right]}^{\prime }}{C_{1}} \end{array}$$

we see:

$$\begin{array}{l} \;R_{0}(\phi )=\frac{1}{2\pi }\\ {\left[R_{1}(\phi )e^{D\phi }\right]}^{\prime }=Q_{1}(\phi )e^{\;D\phi } \end{array}$$

We can use numerical methods to get the solution of *R*_1_(ϕ) given *R*_0_(ϕ) and for some choices of PRCs we can get exact expressions. For example, for the two term double sinusoidal form PRC (Equation 63) we get:

(71) $$R_{1}(\phi )=\frac{1}{C_{1}}\left\{\frac{\tau }{\tau^{2}+1}\frac{\cos(\phi +a_{2}-a_{1})-D\sin(\phi +a_{2}-a_{1})}{1+D^{2}}+\frac{2b_{1}b_{2}\tau }{4\tau^{2}+1}\frac{2\cos(2\phi )-D\sin(2\phi )}{4+D^{2}}\right\}$$

### Different PRCs

#### Double sines form PRC

For the PRC:

$$\Delta_{m}(\theta )=\sin(a_{m})-\sin(\theta +a_{m})+b_{m}\sin(2\theta )$$

We have:

(72) $$\begin{array}{l} h_{mn}(s)=\int_{0}^{2\pi }\Delta_{m}(\theta )\Delta_{n}(\theta +s)d\theta \\ \;=2\pi \sin(a_{m})\sin(a_{n})+\pi \cos(s-a_{m}+a_{n})+b_{m}b_{n}\pi \cos(2s) \end{array}$$

(73) $$\begin{array}{l} g_{mn}(\phi )=\int_{0}^{\infty }h_{mn}(s+\phi )e^{-\frac{s}{\tau }}ds\\ \;=2\pi \tau \sin(a_{m})\sin(a_{n})+\pi \tau \frac{\cos(\phi +a_{n}-a_{m})-\tau \sin(\phi +a_{n}-a_{m})}{\tau^{2}+1}+b_{m}b_{n}\pi \tau \frac{\cos(2\phi )-2\tau \sin(2\phi )}{4\tau^{2}+1} \end{array}$$

(74) $${g^{\prime }}_{mn}(\phi )=-\pi \tau \frac{\tau \cos(\phi +a_{n}-a_{m})+\sin(\phi +a_{n}-a_{m})}{\tau^{2}+1}-2b_{m}b_{n}\pi \tau \frac{2\tau \cos(2\phi )+\sin(2\phi )}{4\tau^{2}+1}$$

(75) $$\begin{array}{l} g(\phi )=g_{12}(\phi )+g_{21}(-\phi )\\ \;=4\pi \tau \sin(a_{1})\sin(a_{2})+2\pi \tau \frac{\cos(\phi +a_{2}-a_{1})}{\tau^{2}+1}+2b_{1}b_{2}\pi \tau \frac{\cos(2\phi )}{4\tau^{2}+1} \end{array}$$

(76) $$\begin{array}{l} g^{\prime }(\phi )={g^{\prime }}_{12}(\phi )-{g^{\prime }}_{21}(-\phi )\\ \;=-2\pi \tau \frac{\sin(\phi +a_{2}-a_{1})}{\tau^{2}+1}-4b_{1}b_{2}\pi \tau \frac{\sin(2\phi )}{4\tau^{2}+1} \end{array}$$

(77) $$C_{1}=g_{11}(0)+g_{22}(0)=2\pi \tau (\sin^{2}(a_{1})+\sin^{2}(a_{2}))+\frac{2\pi \tau }{\tau^{2}+1}+\frac{(b_{1}^{2}+b_{2}^{2})\pi \tau }{4\tau^{2}+1}$$

(78) $$C_{2}={g^{\prime }}_{11}(0)-{g^{\prime }}_{22}(0)=\frac{4\pi \tau^{2}(b_{2}^{2}-b_{1}^{2})}{4\tau^{2}+1}$$

#### Exponential-sine form PRC

For empirical PRCs:

$$\begin{array}{l} \Delta_{1}(\theta )=a_{1}[\sin(b_{1})-\sin(b_{1}+\theta )]e^{c_{1}(\theta -2\pi )}\\ \Delta_{2}(\theta )=a_{2}[\sin(b_{2})-\sin(b_{2}+\theta )]e^{c_{2}(\theta -2\pi )}\\ \theta \in (0,\;2\pi ) \end{array}$$

We have:

(79) $$h_{mn}(s)=B_{1}\;\cdot \;e^{-c_{m}s}(hc_{1},\;hv_{1}(s))+B_{0}\;\cdot \;e^{c_{n}s}(hc_{0},\;hv_{0}(s))$$

(80) $$g_{mn}(\phi )=C_{0}\;\cdot \;e^{\frac{\phi }{\tau }}-e^{-c_{m}\phi }(gc_{1},\;gv_{1}(\phi ))-e^{c_{n}\phi }(gc_{0},\;gv_{0}(\phi ))$$

Where *s* ∈ [0, 2π) and ϕ ∈ [0, 2π), both have the period of 2π. Note also that **(,)** means the inner product of two vectors. The above quantities are defined as:

$$\begin{array}{l} B_{1}=a_{m}\;\cdot \;a_{n}\;\cdot \;e^{-2\pi c_{n}}\;\cdot \;[1-e^{-2\pi c_{m}}]\\ B_{0}=a_{m}\;\cdot \;a_{n}\;\cdot \;[1-e^{-2\pi c_{n}}]\\ D_{1}=c_{m}+\frac{1}{\tau }\\ D_{0}=c_{n}-\frac{1}{\tau }\\ k_{0}=\frac{\sin(b_{m})\;\cdot \;\sin(b_{n})}{c_{m}+c_{n}}-\frac{\sin(b_{m})}{1+{\left(c_{m}+c_{n}\right)}^{2}}[(c_{m}+c_{n})\;\cdot \;\sin(b_{n})-\cos(b_{n})]\\ k_{1}=\frac{1}{2(c_{m}+c_{n})},\;k_{2}=\frac{1}{4+{\left(c_{m}+c_{n}\right)}^{2}},\;k_{3}=-\frac{c_{m}+c_{n}}{2[4+{\left(c_{m}+c_{n}\right)}^{2}]}\\ k_{4}=\frac{\left(c_{m}+c_{n})\;\cdot \;\sin(b_{n}\right)}{1+{\left(c_{m}+c_{n}\right)}^{2}},\;k_{5}=\frac{\sin(b_{n})}{1+{\left(c_{m}+c_{n}\right)}^{2}}\\ j_{0}=\frac{\sin(b_{m})\;\cdot \;\sin(b_{n})}{c_{m}+c_{n}}-\frac{\sin(b_{n})}{1+{\left(c_{m}+c_{n}\right)}^{2}}[(c_{m}+c_{n})\;\cdot \;\sin(b_{m})-\cos(b_{m})]\\ j_{1}=\frac{1}{2(c_{m}+c_{n})},\;j_{2}=-\frac{1}{4+{\left(c_{m}+c_{n}\right)}^{2}},\;j_{3}=-\frac{c_{m}+c_{n}}{2[4+{\left(c_{m}+c_{n}\right)}^{2}]}\\ j_{4}=-\frac{\left(c_{m}+c_{n})\;\cdot \;\sin(b_{m}\right)}{1+{\left(c_{m}+c_{n}\right)}^{2}},\;j_{5}=\frac{\sin(b_{m})}{1+{\left(c_{m}+c_{n}\right)}^{2}}\\ hc_{1}=[k_{0},\;k_{1},\;k_{2},\;k_{3},\;k_{4},\;k_{5}]\\ hc_{0}=[j_{0},\;j_{1},\;j_{2},\;j_{3},\;j_{4},\;j_{5}]\\ hv_{1}=[1,\;\cos(s+b_{n}-b_{m}),\;\sin(s-b_{m}-b_{n}),\;\cos(s-b_{m}-b_{n}),\;\sin(s-b_{m}),\;\cos(s-b_{m})]\\ hv_{0}=[1,\;\cos(s+b_{n}-b_{m}),\;\sin(s+b_{m}+b_{n}),\;\cos(s+b_{m}+b_{n}),\;\sin(s+b_{n}),\;\cos(s+b_{n})]\\ gc_{1}=\frac{B_{1}}{1+D_{1}^{2}}[-\frac{1+D_{1}^{2}}{D_{1}}k_{0},\;-D_{1}k_{1},\;k_{1},\;k3-D_{1}k_{2},\;-k_{2}-D_{1}k3,\;k5-D_{1}k_{4},\;-k4-D_{1}k_{5}]\\ gc_{0}=\frac{B_{0}}{1+D_{0}^{2}}[\frac{1+D_{0}^{2}}{D_{0}}j_{0},\;D_{0}j_{1},\;j_{1},\;j3+D_{0}j_{2},\;-j_{2}+D_{0}j3,\;j5+D_{0}j_{4},\;-j4+D_{0}j_{5}]\\ gv_{1}(\phi )=[1,\;\cos(\phi +b_{n}-b_{m}),\;\sin(\phi +b_{n}-b_{m}),\;\sin(\phi -b_{m}-b_{n}),\;\cos(\phi -b_{m}-b_{n}),\;\sin(\phi -b_{m}),\;\cos(s-b_{m})]\\ gv_{0}(\phi )=[1,\;\cos(\phi +b_{n}-b_{m}),\;\sin(\phi +b_{n}-b_{m}),\;\sin(\phi +b_{m}+b_{n}),\;\cos(\phi +b_{m}+b_{n}),\;\sin(\phi +b_{n}),\;\cos(s+b_{n})]\\ C_{0}=\frac{e^{-2\pi \tau }}{1-e^{-\frac{2\pi }{\tau }}}[(e^{-2\pi c_{m}}-1)\;\cdot \;(gc_{1},\;gv_{1}(0))+(e^{2\pi c_{n}}-1)\;\cdot \;(gc_{0},\;gv_{0}(0))] \end{array}$$

#### Fourier form PRC

For the Fourier form of the PRC:

$$\Delta_{m}(\theta )=\sum_{k=-\infty }^{\infty }a_{m,\;k}e^{ik\theta }$$

(81) $$\begin{array}{l} h_{mn}(s)=\int_{0}^{2\pi }\Delta_{m}(\theta )\Delta_{n}(\theta )ds\\ \;=\sum_{k_{1},\;k_{2}}a_{m,\;k_{1}}a_{n,\;k_{2}}\int_{0}^{2\pi }e^{ik_{1}\theta }e^{ik_{2}(\theta +s)}d\theta \\ \;=2\pi \sum_{k=-\infty }^{\infty }a_{m,\;k}a_{n,\;-k}e^{-iks} \end{array}$$

(82) $$\begin{array}{l} g_{mn}(\phi )=\int_{0}^{\infty }h_{mn}(s+\phi )e^{-\frac{s}{\tau }}ds\\ \;=2\pi \sum_{k=-\infty }^{\infty }a_{m,\;k}a_{n,\;-k}\int_{0}^{\infty }e^{-ik(s+\phi )}e^{-\frac{s}{\tau }}ds\\ \;=2\pi \sum_{k=-\infty }^{\infty }\frac{a_{m,\;k}a_{n,\;-k}}{k^{2}+\frac{1}{\tau^{2}}}(\frac{1}{\tau }-ik)e^{-ik\phi } \end{array}$$
